# Quasi-instantaneous materials processing technology via high-intensity electrical nano pulsing

**DOI:** 10.1038/s41598-023-50698-w

**Published:** 2024-01-03

**Authors:** Eugene A. Olevsky, Runjian Jiang, Wenwu Xu, Andrii Maximenko, Thomas Grippi, Elisa Torresani

**Affiliations:** 1 Department of Mechanical Engineering, San Diego State University, San Diego, 92182 USA; 2 Department of NanoEngineering, University of California San Diego, La Jolla, 92093 USA; 3 Department of Mechanical and Aerospace Engineering, University of California San Diego, La Jolla, 92093 USA

**Keywords:** Metals and alloys, Mechanical engineering

## Abstract

Despite many efforts, the outcomes obtained with field-assisted processing of materials still rely on long-term coupling with other electroless processes. This conceals the efficacy and the intrinsic contributions of electric current. A new device utilizing electrical nano pulsing (ENP) has been designed and constructed to bring quasi-instantaneous modifications to the micro- and nano-structure in materials. Featuring ultra-high intensity (~ 10^11^ A/m^2^) and ultra-short duration (< 1 μs), the ENP technology activates non-equilibrium structural evolutions at nanometer spatial scale and nanosecond temporal scale. Several examples are provided to demonstrate its utility far outpacing any conventional materials processing technology. The ENP technology gives a practical tool for exploring the intrinsic mechanism of electric-field effects and a pathway towards the rapid industrial manufacturing of materials with unique properties.

## Introduction

One of the permanent missions for materials science community is to persistently improve the microstructure of various material systems and obtain expected mechanical or functional properties required by specific application scenarios^1–3^. Given this, a steady stream of research has been emerging for the development of innovative processing technologies, such as field-assisted sintering^4^, ionic liquids^5^, ion beam modification^6^, 3D printing^7^, surface modification^8^, etc. Conventional thermal treatment is still showing impressive potential for properties improvement^9^, phase regulation^10^, grain refinement^11^, etc. However, the long processing duration is a particularly problematic concern in the exploration of advanced treatment technologies. The physical field-involved treatment techniques are appealing in achieving the alteration of surface morphology of materials, like the inorganic layer on electrolyte surface by plasma electrolytic oxidation^12^ and the textured thin layer on metal surface by direct laser interference lithography^13^. However, few breakthroughs have been made on controls at deeper spatial scale or shorter time scale and they are generally material specific.

As a ubiquitous physical phenomenon both in natural and artificial environments, electric field has been employed in many modern engineering fields to develop high-performance materials with unique functional and structural properties. Active research on electrically assisted treatment of solid materials started from the discovery of electroplastic effect^14^. Later, fast-sintering technologies were proposed for powder materials manufacturing, such as flash sintering (FS)^15^, ultra-fast high temperature sintering (UHS)^16^ and spark plasma sintering (SPS)^4,17^. Typical electric field-assisted material processing techniques are generally benefiting from high heating rate and high temperature due to Joule-heating effect. Nevertheless, the pulsed electric current, a type of instantaneous current whose amplitude changes suddenly and periodically over time, is characterized by its distinctive electric stimulus on altering the microstructure and properties of parts^4,18,19^. Compared with continuous electric current, millisecond electric pulses can produce better electroplastic behavior by activating the dislocation reconfiguration in Ti–Al alloy, thereby increasing its tensile elongation and maximum strength^20^. The reduced critical nucleation radius by sub-millisecond electric pulses can generate recrystallization behavior and better plasticity in pure nickel wires subjected to thermomechanical torsion deformation^21^. The industrial-scale application of SPS equipment has also proven the success of millisecond-level electric pulse in promoting the densification of porous body^4^. Beyond these, microsecond electric pulses^22^ contribute to the microstructure refinement and strengthening in cast TC4 titanium-based alloy by promoting the formation of new β phase morphology during thermal-hydrogen treatment^23^. Due to the accelerated hydrogen diffusion by introducing electrical free energy, microsecond electric pulses can even be employed for the de-hydrogenation in steels and their ductility recovery under tensile deformation^24,25^. Furthermore, low-intensity nanosecond electric pulses can find their biological applications in the modulation of cytoplasmic ion concentration of cancerous cells^26^ and the electric excitation of skin neurons^27^. In fact, electric pulsing processing of conductive materials is often combined with other electroless processes, rather than being treated as a stand-alone treatment. This may complicate the argument that the conventional processes, such as mechanical deformation or chemical diffusion, can produce synergistically enhanced or destructive outcomes when combined with electrical pulses. Although the field effect is reported to be more significant with more intense electric field^28,29^, the intrinsic influences of electrical pulses on microstructural modifications are difficult to intuitively highlight in this case. Moreover, short-duration electric pulse with the ON-phase period down to milli- or microseconds has been widely applied to many conductive material systems, but the entire process often does not last on the same but a much longer time scale^30^. Single or multiple low-intensity electrical pulses often fail to trigger the anticipated modification results, whereas the detectable outcomes rely on the energy accumulation from massive low-frequency electric bursts. This becomes an obstacle to the reliable high-throughput materials processing unless high current intensity, high frequency and short pulse duration can be acquired simultaneously in the electric pulsing technology. Therefore, constructing the high-energy electric micro-nano pulsing technology with desirable output characteristics and utilizing its thermal and/or electric field effects for quasi-instantaneous materials processing should exhibit attractive scientific significance and industrial potential.

Here we introduce a unique electric pulsing treatment method based on the fast capacitor discharging technique, called electric nano pulsing (ENP) technology, featuring ultra-high voltage (up to 1000 V), ultra-high current density (up to 10^11^–10^12^ A/m^2^), ultra-short pulse duration (less than 1 μs) and ultra-high pulsing frequency (up to 100 kHz). The high intensity impulse current released into polycrystalline materials within the micro-nano seconds enable the extremely rapid Joule heating effect and the ultra-strong electric field effect, thereby having the ability to activate the non-equilibrium structural evolutions of both grain boundaries (GBs) and grain insides (GIs) at nanometer spatial scale and nanosecond temporal scale. This is far more advanced than existing conventional electric pulsing treatment.

## Materials and methods

### The design and performance of ENP device

**Figure 1:**
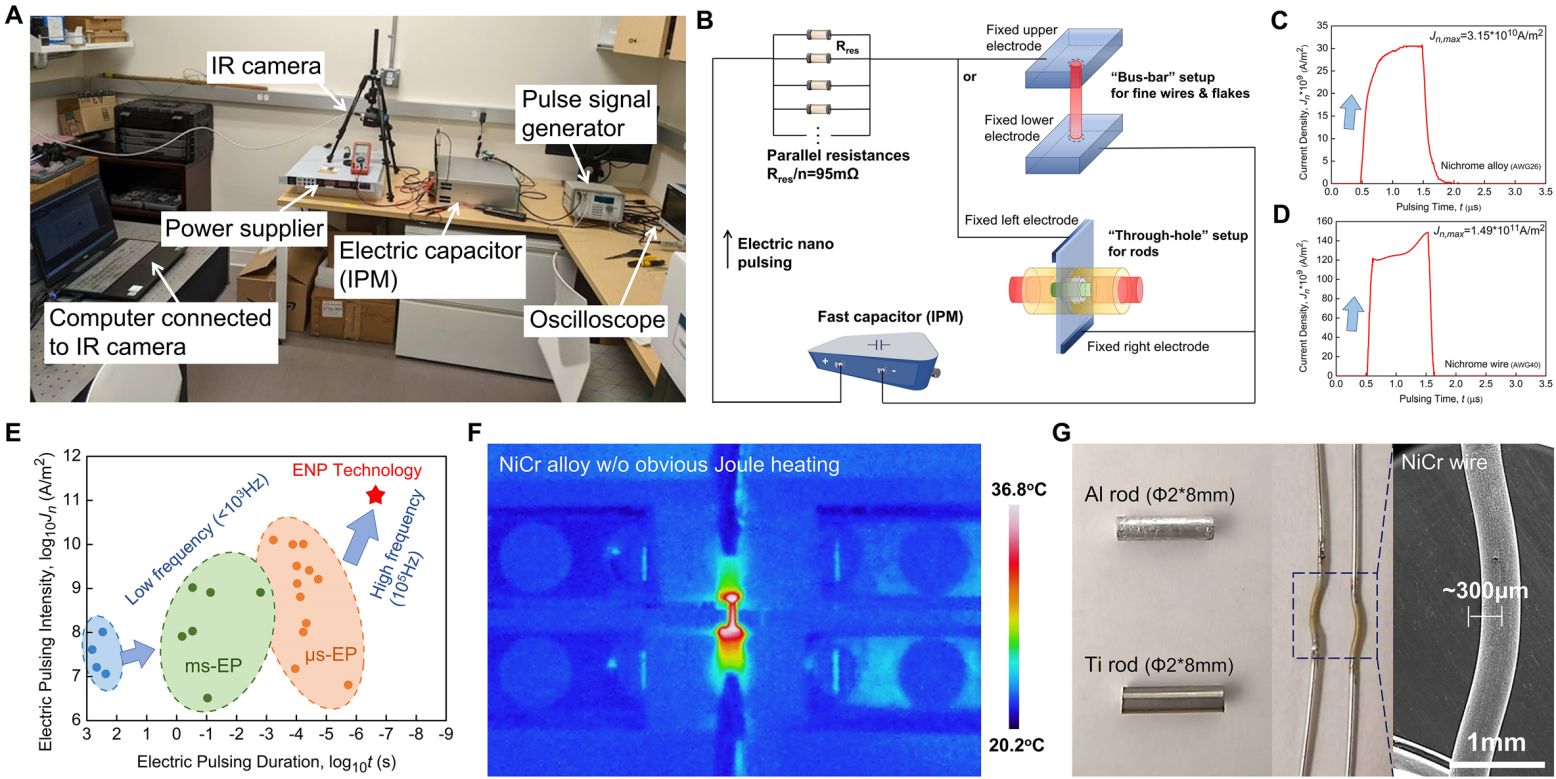
The illustration of proposed electric nano pulsing (ENP) technology. (**A**) Physical picture of the constructed ENP machine with all major component labeled. (**B**) Schematic diagram showing the electric circuit features and possible sample set up in ENP machine. (**C**) Example on the electric pulsing showing the current density curve (3.15 × 10^10^ A/m^2^) output by ENP machine. (**D**) Another example on the electric pulsing showing the current density curve (1.49 × 10^11^ A/m^2^) output by ENP machine. (**E**) Comparison between the proposed ENP technology and other existed electric pulsing technologies showing all-around performance improvement^14,20,21,23–25,31–47^. (**F**) Example on the temperature map of nichrome alloy during ENP process showing no obvious temperature rise by Joule heating effect. (**G**) Exemplary images of some conductive materials that are subject to ENP process by different sample setups.

The schematics of the ENP device is shown in Fig. 1A, with a diagram (Fig. 1B) briefly clarifying its electric circuit features. The DC power supplier enables the energy storage of integrated power module (IPM) through high voltage electricity up to 1000 V, and the energy release can be triggered by pulse signal generator outputting the ultra-short square wave signal ranging from 100 ns to a few hundreds of microseconds. The flexibly customized design of IPM (EHT Inc., Seattle, USA) mainly contains fast capacitors with solid-state switch, which allows the achievement of ultra-short rise or fall time of a high-intense electric current pulsing. In the ENP device, a group of parallel constant resistances (~ 95 mΩ) are embedded on printed circuit board (PCB) board for over current protection, and its total inductance is 22.5 nH. Two types of sample setups are designed for experimentations, named “bus-bar” and “through-hole” respectively. The fine wires or thin flakes can be attached directly to the fixed upper/lower electrodes 3.5 mm apart in “bus-bar” setup; alternatively, the rods can be connected to the fixed electrodes distributed on both sides of the PCB by the pressure exerted by the soldered fasteners in “through-hole” setup. Given that the ENP device exhibits great flexibility in the sample-electrode configurations, materials processing for both powders and bulks is highly possible for various practical purposes with the support of IPM component. The proposed ENP technology in this work has an intensity 10^1^–10^2^ times stronger than other existing electric pulsing techniques, whilst simultaneously has a distinctive nanosecond level pulsing duration^14,20,21,23–25,31–47^. With the fast-discharging technology, the current density in the ENP device can be excited to hundreds of billions of Amps per square meter within 100 ns, based on the front edge of output pulsing signals in Fig. 1C and D. Its reliability is thus guaranteed by the capability of providing precise and stable ultra-short current pulsing. The seconds-level EP techniques are often unattractive with very long duration and low intensity. Although milli-EP and micro-EP technologies have seen improvements in current intensity, they still have not gotten rid of the low frequency limit (generally much less than 10^3^ Hz) (Fig. 1E). On the premise of improving current intensity, the ENP technology has further increased the pulse frequency by two orders of magnitude, reaching an extraordinary 10^5^ Hz. These excellent features can also inspire a whole new concept of material processing, namely, the ENP-assisted instantaneous modification of localized material microstructures (surface, GBs or GIs). The principle of ENP processing is that quasi-instantaneous materials processing can be achieved by applying the high frequency electric pulsing with ultra-high current intensity and ultra-short pulsing duration. The ultra-high current intensity is in favor of emphasizing the contribution of field effects, whilst the ultra-short pulsing duration ensures the availability of quasi-instantaneous processing and avoids the negative effects of excessive Joule heating. Figure 1D and G display some images of exemplary materials during or after ENP processing. Apparently, the proposed ENP technology is capable of both the electrically stimulated treatment of materials under short-duration pulses and the rapid thermal treatment under high-frequency pulses. The resulting non-equilibrium microstructural evolution in the localized regions of materials and the accompanying other phenomena possibly can be drastically enhanced compared to the conventional techniques, which are the attractive scientific topics in the future.

### Materials and experimental procedure of ENP processing

The commercial AWG26 Ni-based superalloy wire (softening annealed; 80Ni–18.5Cr–1.5Si in wt%; D = 403.9 μm; FCC lattice with a = 0.3539 nm) is used as target material. The quasi-instantaneous processing on both localized surface and interior of this superalloy is explored based on the ENP technology. Ni-based superalloys with good mechanical performance and oxidation resistance are one of the most extensively studied superalloys and have wide industrial applications at high temperature environments^48^. This Ni–Cr–Si superalloy wire has good electric conductivity (84 mΩ/cm) at room temperature and low temperature coefficient of resistance (8.5 × 10^–5^/K), which ensure it to be a suitable material in ENP processing. A 12 mm-length Ni–Cr–Si superalloy wire was installed in the “bus-bar” setup, with alumina ceramic flake pressed onto it for ideal electrode-sample contact. The total resistance of the whole sample setup is measured to be ~ 80.5 mΩ. The fast capacitor was charged at an input voltage of 600 V. The high-intensity electric pulsing with an input signal duration of 1 μs and an ON-to-OFF pattern of 1:9 (10% duty cycle) was released into the wire in the air atmosphere. Therefore, one pulsing signal was sent to the target material every 10 μs, and the pulse frequency is 100 kHz accordingly. A total of eight pulses were generated and the whole processing time was 80 μs. Once the electric pulses were triggered, the entire processing could happen suddenly. The current density profile of one of output pulsing signals is shown in Fig. 1C. The peak current density along the wire can reach 3.15 × 10^10^ A/m^2^, which is sufficiently high to bring instantaneous modification to the material microstructure. Another ENP processing with a total of two pulses at a frequency of 100 kHz and a peak current density of 6.98 × 10^10^ A/m^2^ was conducted as well. In this work, the rationale for selecting those parameters is that under the premise of ensuring short duration and high intensity, ultra-fast processing can be applied to make the temperature of this superalloy exactly reach its melting point. The estimation of the skin depth in this pulsing condition (~ 523 μm) indicates a uniform current and temperature distributions across the wire cross-section. This estimation is confirmed in the next section by modeling results of the temperature distribution in this wire. For comparison, the conventional heating of identical nichrome wires was conducted by placing them into an atmospheric furnace preset at 1400 °C (melting point) for 10 s. The capability of the ENP technology goes beyond the rapid surface modification, whilst its role in tailoring the GBs morphology and the GIs defects should not be underestimated^49^. Here we provide some straightforward examples on the quasi-instantaneous localized processing of the surface thin oxidation coating as well as the internal dislocation hardening in this conductive material.

### Modeling of temperature field on Nichrome alloy during ENP processing

The uniformity of the heating depends on the magnitude of the skin effect, which, in turn, is a function of the shape and periodicity of electric pulses. Experimental measurement of the temperature evolution during electrical nano pulsing is a significant challenge. In our experiments, temperature was estimated numerically based on the known electrical current pulse shapes and duty cycle. The shape of the electrical current pulses used in this experiment with nichrome wire is shown in Fig. 1C. The evolution of electric field strength across the wire cross-section follows the equation^50^:

1 $$ \left\{ {\frac{1}{\rho }\frac{\partial }{\partial \rho }\rho \frac{\partial }{\partial \rho }} \right\}E\left( {\rho ,\tau } \right) = \frac{\partial }{\partial \tau }\left( {\frac{{\sigma \left( {T,\tau } \right)}}{{\sigma_{0} }}E\left( {\rho ,\tau } \right)} \right) $$

where $\rho = r/R$ and $\tau = t/\tau_{0}$ are dimensionless variables. Here $\tau_{0} = R^{2} \mu_{0} \sigma_{0}$ could be treated as the characteristic time of skin effect, which is equal to 4 × 10^−8^ s in this case. $r$ is the radial coordinate in the circular section of nichrome wire with the radius equal to $R$, and $t$ is the time. $\mu_{0}$ and $\sigma_{0}$ are the magnetic permeability and the conductivity of nichrome alloy at room temperature, correspondingly. The boundary condition for this equation is obtained because of continuity of the electric field strength through the wire boundary^50^:

2 $$ \frac{\partial E}{{\partial \rho }}\left( {\rho = 1} \right) = \frac{{\mu_{0} }}{2\pi }\dot{I} $$

where $\dot{I}$ is the time derivative of the total electrical current through the wire, which can be calculated from the current pulse configuration (as shown in Fig. 1C). The electrical current density across the wire cross-section can be related to the electrical field strength by the Ohm’s law:

3 $$ J = \sigma E $$

All material parameters of this nichrome alloy that are used in these calculations are given in Table 1^51,52^. The direct finite-difference calculations of Eq. (1) have confirmed that the electric current during this one microsecond pulse is almost uniform across the wire cross-section. The temperature evolution across the wire cross-section is described by traditional thermal diffusion equation. With the use of dimensionless parameters, it has the following form^50^:

4 $$ \frac{{\rho_{m} C_{p} }}{{\tau_{0} }}\left[ {\frac{\partial T}{{\partial \tau }} - D\frac{\partial }{\partial \rho }\left( {\frac{k\left( T \right)}{{k_{0} }}\frac{\partial T}{{\partial \rho }}} \right)} \right] = \sigma E^{2} $$

and,

5 $$ D = \frac{{k_{0} \mu_{0} \sigma_{0} }}{{\rho_{m} C_{p} }} $$

where $\rho_{m}$ is the density of nichrome alloy, $C_{p}$ is the heat capacity of nichrome alloy. $k\left( T \right)$ is the thermal conductivity of nichrome alloy and $k_{0}$ is its value at room temperature. Heat irradiation and convection are considered in the boundary condition as following:

6 $$ - k\left( T \right)\frac{\partial T}{{\partial \rho }}\left( {\rho = 1} \right) = \epsilon Rk_{B} \left( {T^{4} - T_{0}^{4} } \right) + Rh\left( {T - T_{0} } \right) $$

where *ϵ* is the emissivity, $k_{B}$ is the Stephan–Boltzmann constant and $T_{0}$ is the ambient temperature. The convective heat transfer coefficient $h$ for the wire can be calculated as^53^:

7 $$ h = \frac{Nu \cdot k\left( T \right)}{{2R}} $$

8 $$ Nu^{\frac{1}{2}} = \frac{{0.6 + \left( {0.387Ra^{\frac{1}{6}} } \right)}}{{\left( {1 + \left( {\frac{0.559}{{Pr}}} \right)^{\frac{9}{16}} } \right)^{\frac{8}{27}} }} $$

where $Ra$ and $Pr$ are the Rayleigh and Prandtl numbers calculated based on the physical parameters of air at room temperature. In this experiment with nichrome wire, eight consecutive one-microsecond pulses were used with the 10% duty cycle. Equations were solved by finite difference method with FORTRAN code. Taking the electric field uniformity into consideration, the temperature evolution and distribution in nichrome wire during this quasi-instantaneous ENP processing can be predicted.

**Table 1:** The electro-thermal properties of nichrome alloy and air used for the calculation of temperature evolution during ENP processing.

	Nichrome alloy	Air
Density, *ρ* (kg m^−3^)	8400	1.29
Thermal capacity, *C*_*p*_ (J kg^−1^ K^−1^)	460	1061.3–0.43282*T* + 1.0234 × 10^−3^*T*^2 ^− 6.4747 × 10^−7^T^3^ + 1.3864 × 10^−10^T^4^
Thermal conductivity, *K*_*a*_ (W m^−1^ K^−1^)	12.375 × (1 + 0.033*T*/298 + 0.00011 × (*T*/298)^2^)	− 7.488 × 10^–3^ + 1.798 × 10^−4^*T* − 2.3578 × 10^−7^*T*^2^ + 2.2012 × 10^−10^*T*^3^ + 9.46 × 10^−14^*T*^4^ + 1.5797 × 10^−17^*T*^5^
Electrical conductivity, *σ* (S m^−1^)	1/(1.1 × 10^–6^ + 1.1 × 10^−10^*T* − 7.0 × 10^−14^*T*^2^)	N/A
Emissivity, *ε*	0.87	N/A
kinematic viscosity, *ν*_*a*_ (m^2^ s^−1^)	N/A	(4.113 × 10^–6^ + 5.0523 × 10^−8^*T* − 1.4346 × 10^−11^*T*^2^ + 2.5914 × 10^−15^*T*^3^)/*ρ*

For air properties, Rayleigh number *Ra* equals to $16g\left( {T_{s} - T_{a} } \right)R^{3} \rho C_{p} /k_{a} \nu_{a} \left( {T_{s} + T_{a} } \right)$, and Prandtl number *Pr* equals to $\nu_{a} \rho C_{p} /k_{a}$, where *T*_*s*_ is the temperature at the wire surface, *T*_*a*_ is the ambient temperature, *R* is gas constant and *g* is convective heat transfer coefficient^51,52^.

## Results

### Quasi-instantaneous and localized processing via ENP technology-temperature field and grain structure stability

**Figure 2:**
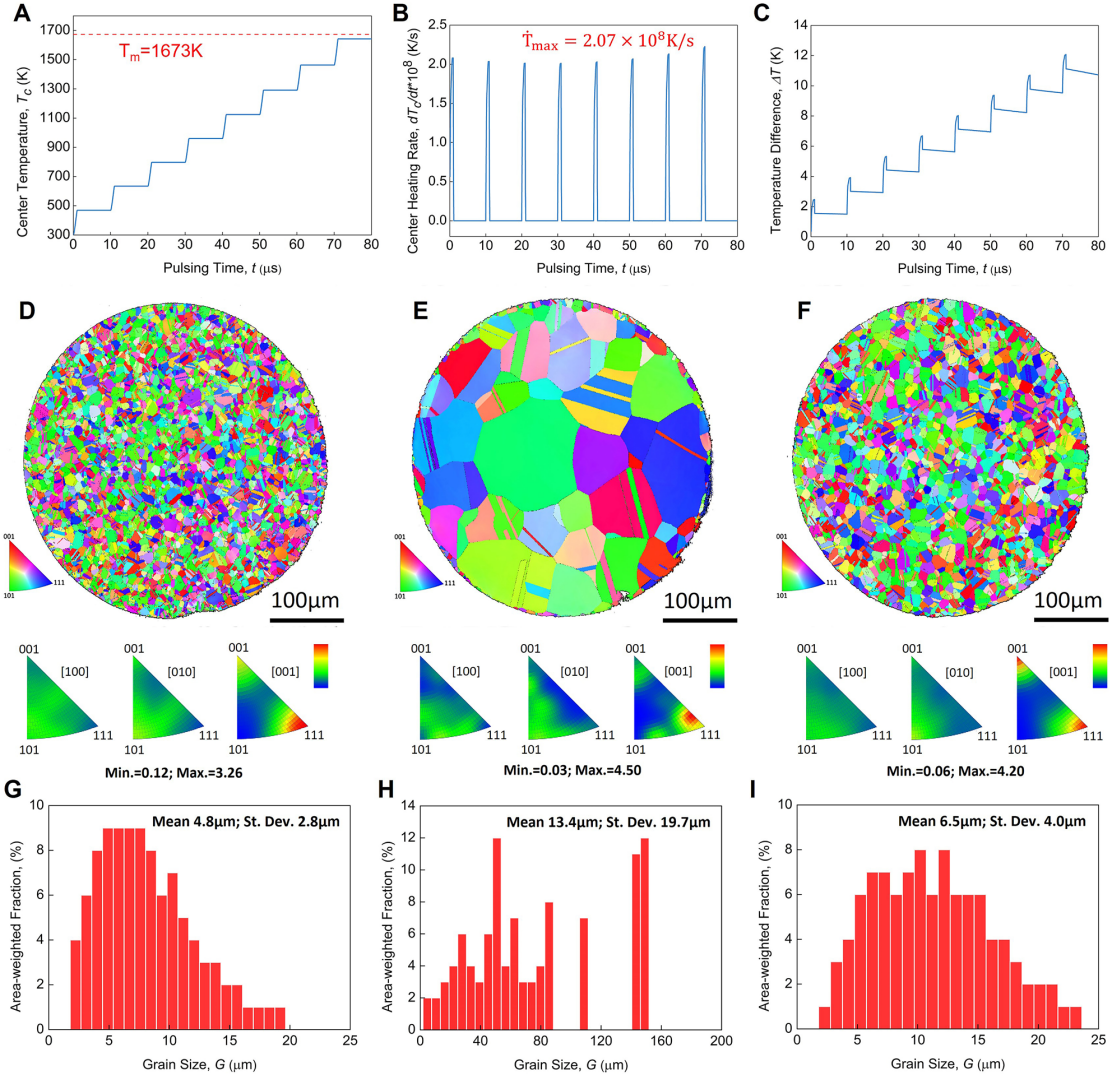
The simulated temperature and measured microstructure of nichrome alloy demonstrating the quasi-instantaneous and localized features of ENP processing. (**A**) The temperature at alloy component’s center over the pulsing time. (**B**) The corresponding heating rate over the pulsing time. (**C**) The temperature different between the alloy component’s center and the edge over the pulsing time. (**D**, **E** and **F**) EBSD inverse pole figures (IPF) of the cross-sections of nichrome wires. (**G**, **H** and **I**) Grain size distributions of nichrome wires: (**D** and **G**) raw sample, (E and H) after conventional heating, (**F** and **I**) after ENP processing.

One of the most common concerns regarding the quasi-instantaneous ENP processing at micro-nano seconds scale is the distribution of electric field and temperature within the treated material component’s cross-section, which, to large extent, determines whether this proposed ENP technology can be compatible with industrial applications. By simulation, the rise of temperature at alloy center exhibits stepped increment in this case (Fig. 2A), with the maximum wire temperature being about 1643 K, which is very close to the melting point of this nichrome alloy in this work (1673 K). This agrees with the experimental observation that releasing extra electric energy will cause the bulk melting of this nichrome alloy. The corresponding temperature rate profile shows that the proposed ENP technology can bring this nichrome alloy an extraordinary heating rate of the magnitude of 10^8^ K/s during the pulsing releasing whilst the cooling during the rest time is negligible (Fig. 2B). It is noted that the temperature difference between the alloy component’s center and the edge, although gradually increasing over the pulse time, can maintain at a sufficiently low value (less than 12 K) (Fig. 2C).

**Figure 3:**
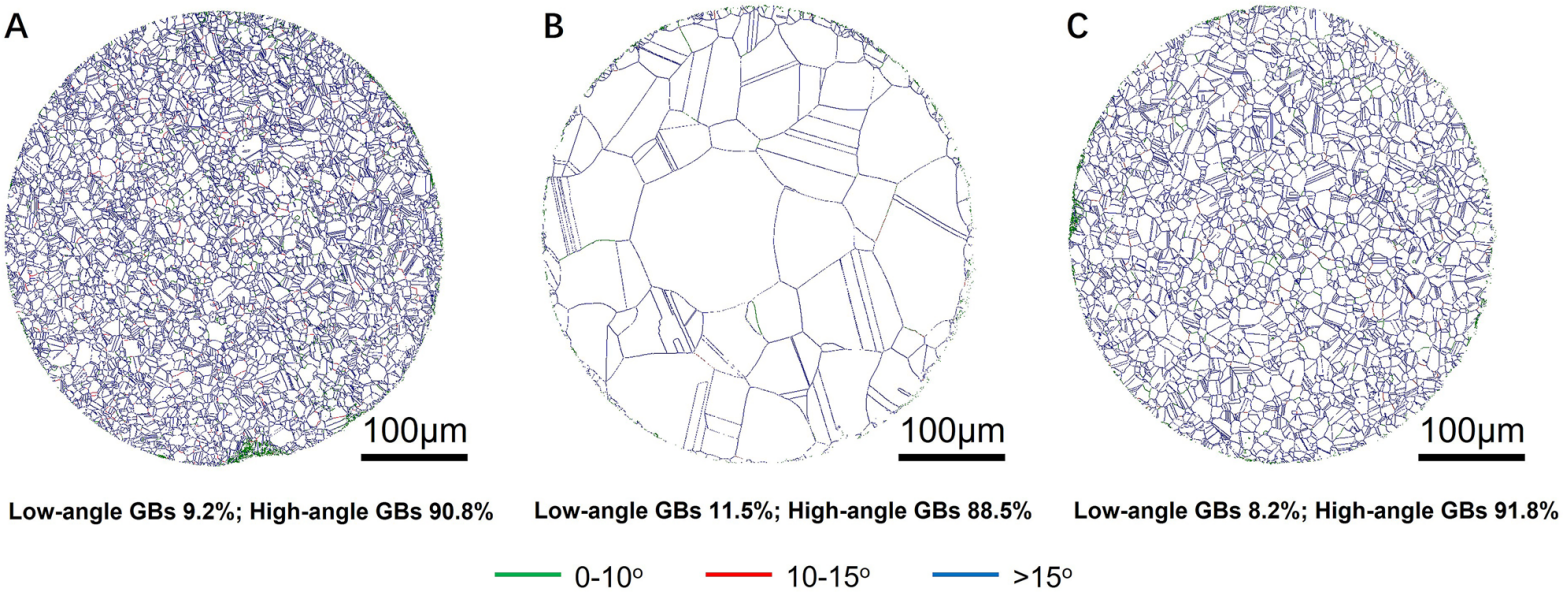
Images showing the grain boundaries distributions and orientations in nichrome alloys. (**A**) Raw sample. (**B**) After conventional heating. (**C**) After ENP processing.

Another important feature of ENP technology is the localized modification of material structure at micro-nano structure level without significant change of grain structure. Figure 2 also shows the EBSD inverse pole figures and the grain size distributions of nichrome alloy wires after conventional heating and after ENP processing. For conventional heating, the nichrome alloy wires were loaded into a conventional furnace preheated to 1400 °C for 10 s to achieve the highest possible heating rate and the shortest possible duration time as close as possible to the condition in ENP processing. The annealing twins are commonly seen in this Ni-based superalloy with FCC structure subjected to thermal treatment^54^. This comparison clearly shows that the grain structure of alloy matrix remains almost unchanged after ENP processing, while the conventional heating brings it a drastic modification. Many thermal processing technologies by heat conduction/radiation or by Joule heating generally require a duration time of at least dozens of seconds or even much longer. One can see from Fig. 2D,E,G,H that the nichrome alloy grains undergo a significant growth in size after only 10 s of conventional heating, with the mean grain size increasing by nearly three times that of the raw sample (from 4.8 to 13.4 µm), and an unexpected maximum size of 146.2 µm. However, compared with conventional heating, no obvious change in grain morphology occurs after the ENP processing, as illustrated by their similar distribution range and mean size (Fig. 2I). The raw nichrome wire has a pronounced grain orientation texture. The <001> and <111> orientations are preferred to align with (001) direction (axial direction of wire), whilst the radial direction have a random orientation distribution (Fig. 2D). This orientation feature, at a smaller structural level, is modified after ENP processing (Fig. 2F) and has been significantly damaged after conventional heating. The grains at radius directions exhibit some preferred orientations, and the closest-packed plane {111} no longer tends to be parallel to the wire cross-section (Fig. 2E). The inspection of the orientation difference at GBs also indicates that the high-angle GBs still hold the majority (> 90%) both in raw sample and after ENP processing, whereas conventional heating promotes the formation of more low-angle GBs (> 10%) (Fig. 3). More emphasis will be put on the quantification of Σ3^n^ special grain boundaries by EBSD data in next works, as this is at the heart of properties optimization via grain boundary engineering (GBE)^55^.

### The quasi-instantaneous ENP processing as defect reconfiguration technology

**Figure 4:**
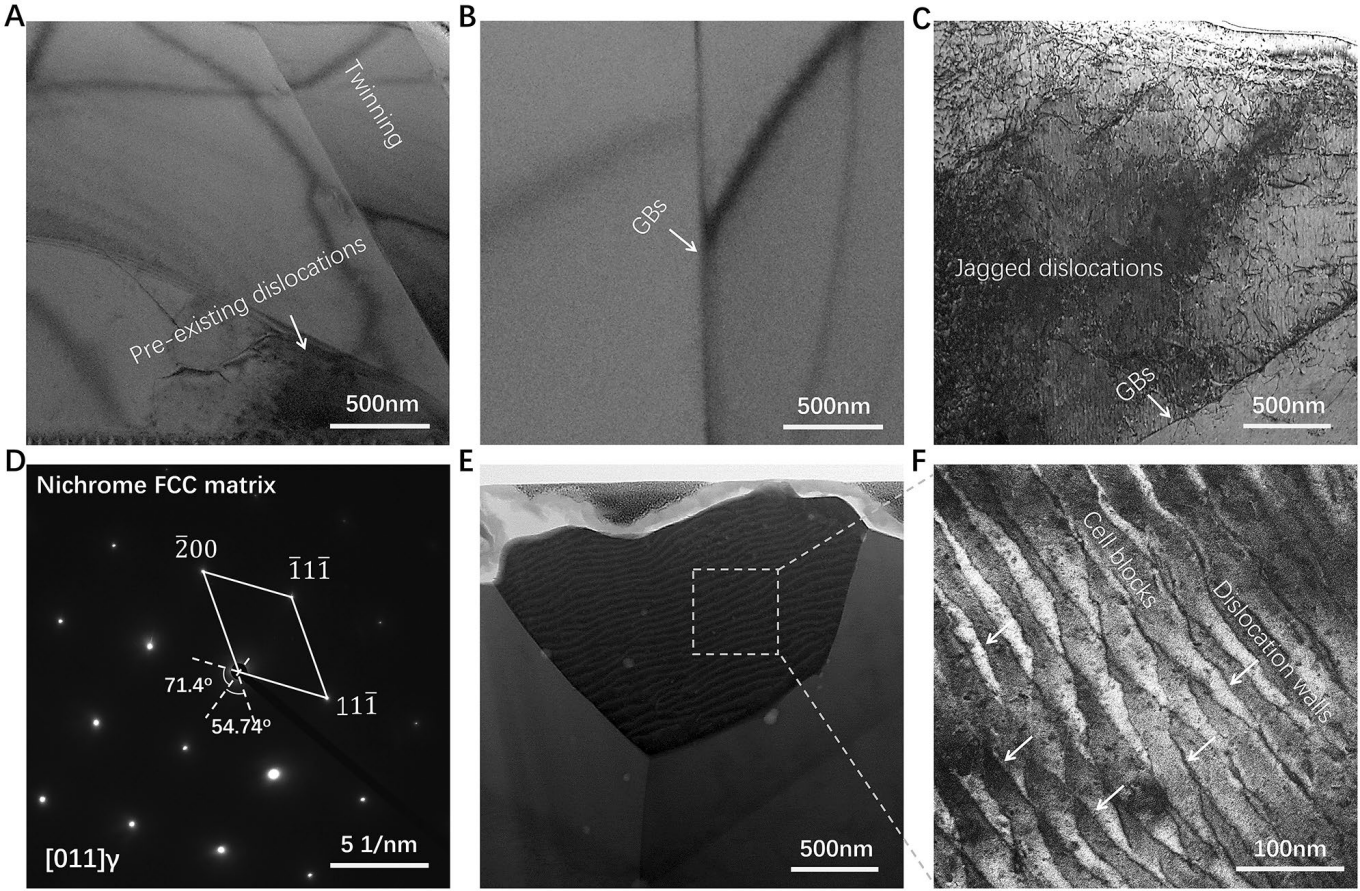
Bright-field TEM images showing the comparison of dislocation configurations in nichrome alloys. (**A**) Raw sample. (**B**) After conventional heating. (**C**) After ENP processing of two electric pulses with current density of 6.98 × 10^10^ A/m^2^, pulsing duration of 1 μs and pulsing frequency of 100 kHz. (**D**) Corresponding selected area electron diffraction pattern (SAED) of the FCC matrix along [011] zone axis. (**E**) After ENP processing of eight electric pulses with current density of 3.15 × 10^10^ A/m^2^, pulsing duration of 1 μs and pulsing frequency of 100 kHz. **(F**) Zoom-in image on the white dash square area showing the detailed dislocation morphology.

The massive energy released into nichrome alloy via ENP processing can also bring the non-equilibrium modifications on material’s internal microstructure within micro- or nanosecond scale. One of representative manifestations is the influence of change of grain insides (GIs) on hardening effects by producing dislocations. It is expected that the intense electric pulsing promotes dislocation motion due to electroplastic effect and, indeed, the wire length after ENP processing is increased due to its plastic deformation. The observations by transmission electron microscopy (TEM) display a dramatic difference in dislocation morphology between raw and ENP processed samples. As shown in Fig. 4A, the raw nichrome alloy has a dislocation entanglement structure produced by plastic deformation. These pre-existing dislocations should be generated by cold rolling of raw nichrome wire and reserved after its softening annealing. As expected, conventional heating near melting point promotes the dislocation mobility and the annihilation of opposite-signed dislocations, thereby reducing the dislocation density significantly (Fig. 4B). Surprisingly, this ENP processing not only promote the avalanche generation of dislocations, but also alternate the dislocation configuration in a dramatic way. Here some representative examples are presented. In ENP processed nichrome wire with two pulses at a current density of 6.98 × 10^10^ A/m^2^, a large number of jagged dislocations with heavily curved and randomly oriented topography can be observed by imaging at (011) zone axis (Fig. 4C), with selected area diffraction pattern shown in Fig. 4D. It probably results from the cross-slip of extended screw dislocations or the point pinning of dislocation lines under this ENP process. Similar dislocation reconfiguration is also found in pulsing deformed Ti–Al alloy, which is described as the homogeneous wavy slip and the source of electroplasticity^20^. It is supposed to harden this ENP processed alloy by suppressing planar slip and increasing work hardening rate. Additionally, in ENP processed nichrome wire with eight pulses at a current density of 3.15 × 10^10^ A/m^2^, a group of dislocation walls with orderly interwoven and periodically arranged topography can be observed by imaging at (011) zone axis (Fig. 4E,F). These dislocation walls are the boundaries that divide the matrix into small cell-like regions (cell blocks) with relatively low density. This is caused by the multiplication and rearrangement of entangled dislocations with respect to the motion direction of drifting electrons in this ENP process. Similar universal parallel distribution of dislocations is also found in quenched steel after electropulsing, which is described as “seaweed” structure formed by electron wind force^56^. It can bring the electroplastic effect by combined action of Joule heating and reduced residual stress. The arguments on the dislocation rearrangement by electropulsing treatment and its resulted electroplasticity are diverse. The participation of Joule heating should be in favor of increased plasticity, but the origin of electroplasticity and its impact on mechanical properties have not been explained consistently^57–59^. It is commonly assumed that electropulsing promotes the dislocation motion by electron wind force, but our results here indicate that different ENP parameters, including current intensity, pulsing duration, and pulsing rising rate^60^, can produce specific mechanism of dislocations-pulsing interaction, finally contributing to the exhibition of various dislocation configurations. The ENP technology can stimulate specific electro-microstructure interaction by precisely controlling the input of this powerful and responsive electric energy in a material system and provide a creative tooling for exploring the mechanism of relevant electric field effects.

**Figure 5:**
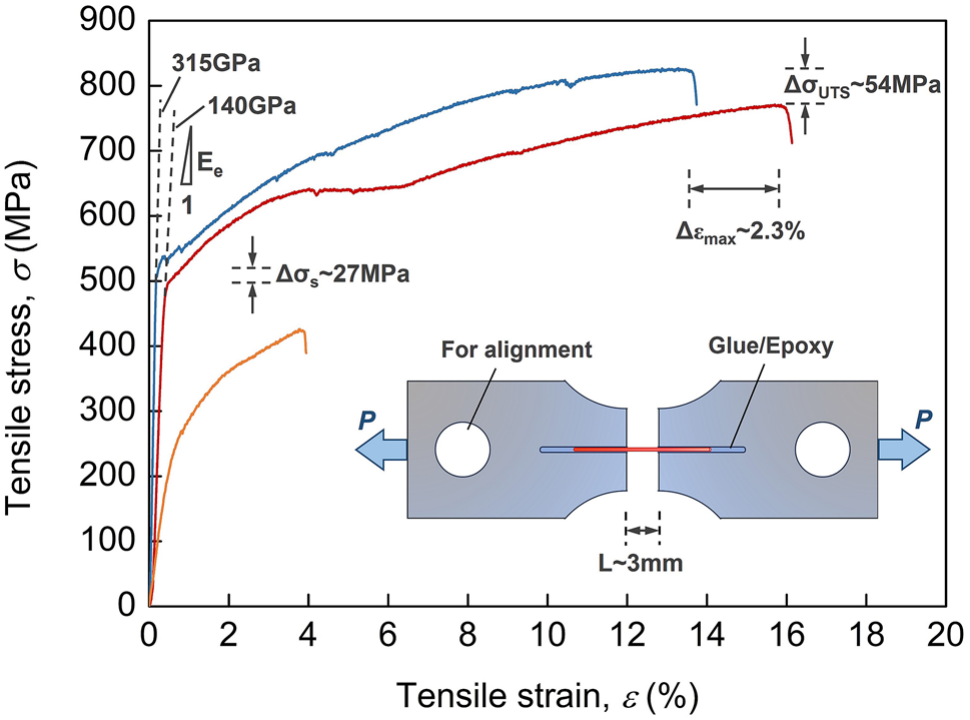
Engineering stress–strain curves of nichrome alloys under tensile test. raw sample (red); after ENP processing (blue) and after conventional heating (orange). The ENP processing has eight electric pulses at the current density of 3.15 × 10^10^ A/m^2^, the pulsing duration of 1 μs and the pulsing frequency of 100 kHz. The insert is a schematic diagram showing the Aluminum setup tooling for tensile test of fine alloy wire at Instron 5800 mechanical testing machine. Cylinder rods are inserted into the round holes and clamped onto the fixtures of Instron machine to obtain good alignment of alloy wires during tensile tests. Glue or epoxy (curing in vacuum) are used to mounting the two ends of alloy wires to achieve good bonding and minimize the stress concentration during tensile tests.

**Figure 6:**
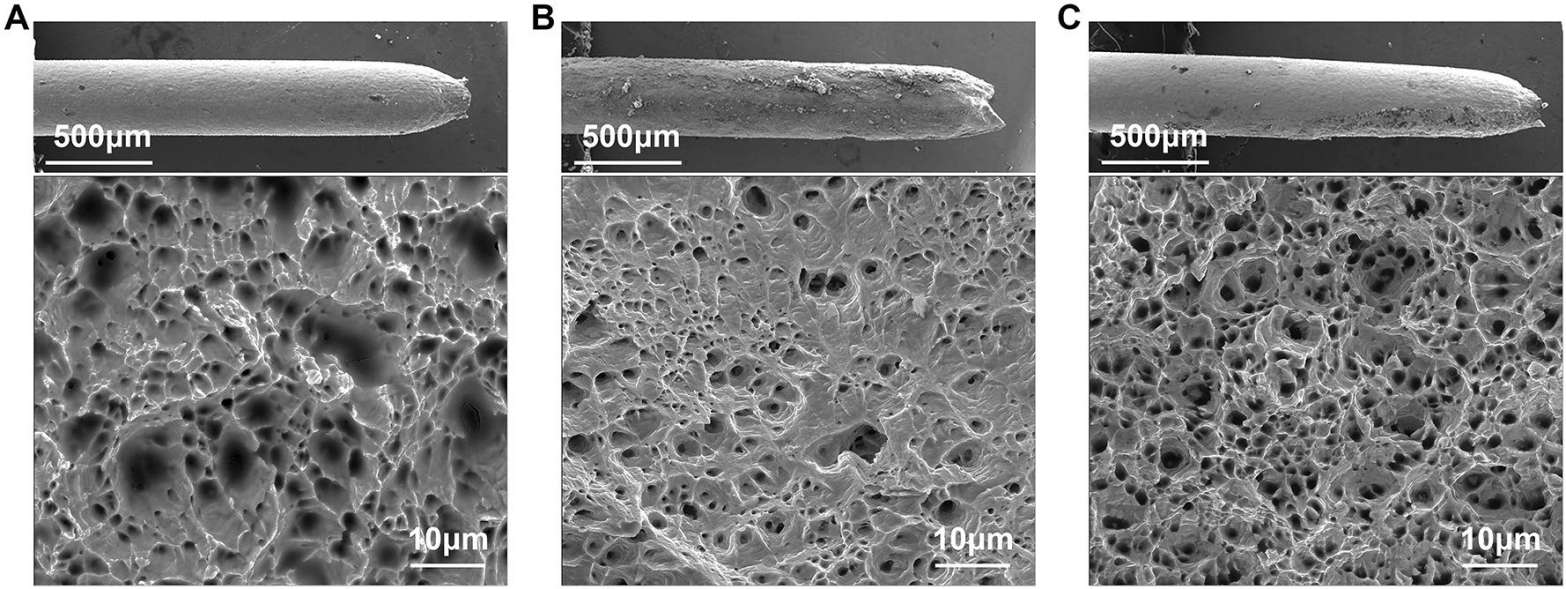
SEM images showing the macroscopic fracture surface and the detail ductile fracture features of nichrome alloy after different processing. (**A**) Raw sample. (**B**) After conventional heating. (**C**) After ENP processing. The ENP processing has the current density of 3.15 × 10^10^ A/m^2^, the pulsing duration of 1 μs and the pulsing frequency of 100 kHz. All the nichrome alloys show typical ductile fracture behavior. The fracture surface of alloy after ENP processing still has the same dense dimple structure as that of raw sample. However, conventional heating produces the loose dimple structure with partial brittle characteristics on its fracture surface, indicating an obvious properties degradation.

Tensile testing of nichrome alloy wires was conducted to analyze the mechanical impact of ENP processing (Fig. 5), with their fracture surfaces shown in Fig. 6. Experimental results show that slight softening phenomenon with a small decrease of ultimate fracture strength is observed in this alloy after ENP processing, despite the dislocation reconfiguration produced by electropulsing (Fig. 5). This can result from the temperature rise by Joule heating, whilst the heavy dislocation generation or reconfiguration is locally happened in a few specific grains. It is inferred that this softening effect on materials can be avoided in ENP processing by suppressing Joule heating. The yield stress does not show a signification change due to the unaffected grain size after ENP processing. Conventional thermal treatment brings this nichrome alloy to an all-round property degradation, because of dislocation annihilation, abnormal grain growth and loose oxide mixture on the surface. Remarkably, the elastic modulus of nichrome alloy after ENP processing changes from 315 to 140 GPa, which indicates an obvious transformation of the initial anisotropic texture in a raw sample produced by cold rolling to the relaxed isotropic structure. Our experiments show almost instant release of the stored mechanical energy by previous cold deformation in the nichrome alloy wire under ENP processing without significant change of its grain structure. This means the localization of electrical pulsing impacts predominantly at the dislocation level. Just by releasing a few electric pulses, their high intensity nature ensures the significant non-thermal effects. Homogenization and uniformization of material properties in Ni-based superalloys during electropulsing treatment have been detected^61^. However, it takes place in micro-nano seconds and without detectable recrystallization in our case. This effect produced by electropulsing can be used for the improvement of material properties^62^.

### The quasi-instantaneous ENP processing as surface nanocoating technology

**Figure 7:**
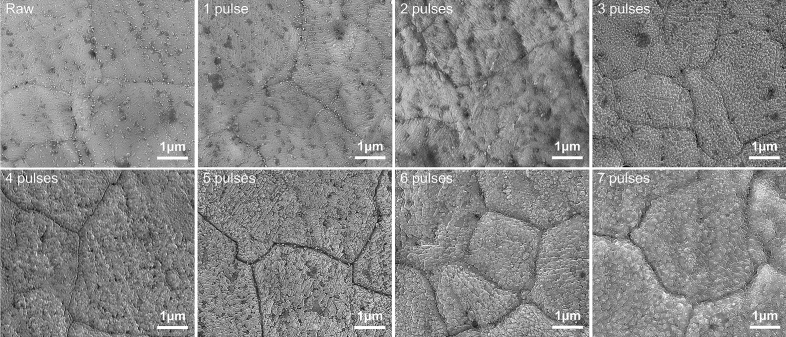
SEM images showing the evolution of exterior surface morphology on the nichrome alloy surface after multiple electric pulsing. This alloy was subjected to ENP processing with the current density of 3.15 × 10^10^ A/m^2^, the pulsing duration of 1 μs, the pulsing frequency of 100 kHz and the duty period of 10%. The triple hierarchical structure of nanocoating on alloy exterior surface is formed after its gradual development during ENP processing. The obvious oxidation formation is seen on alloy surface after releasing three pulses (3 μs pulsing time or 30 μs total processing time). After seven electric pluses are released in this case, only more severe oxidation formation is observed, which indicates that this triple hierarchical structure of nanocoating is instantaneously generated within 1 μs at the temperature near melting point.

**Figure 8:**
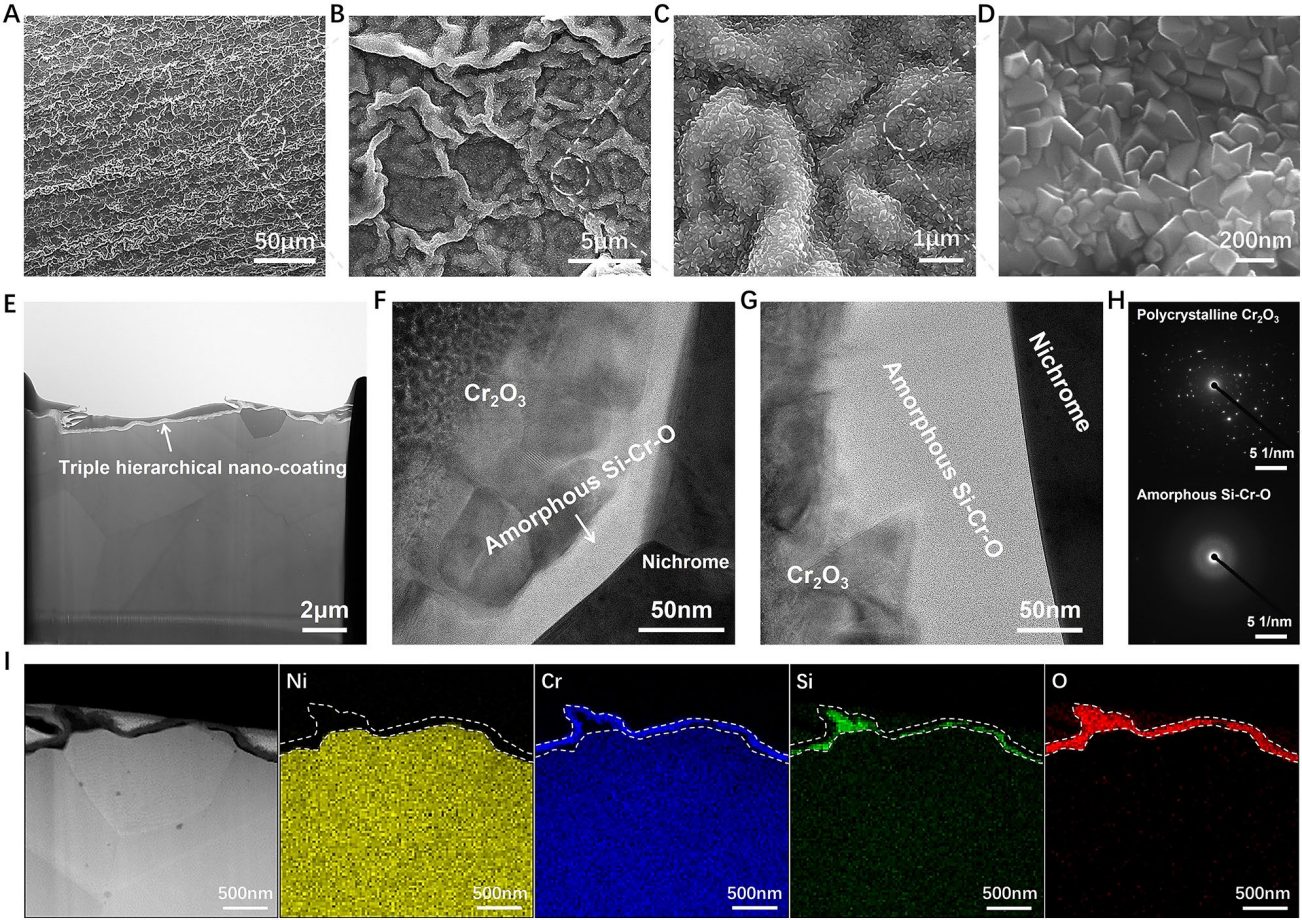
The morphology of nanocoating on nichrome alloy after ENP processing. (**A–D**) The exterior surface morphology by SEM imaging showing the triple hierarchical structure of nanocoating on the nichrome alloy surface: (**A**) low-magnification view, (**B**) fold-like morphology at micro-scale, (**C**) ravine-like morphology at sub-nanoscale and (**D**) well-faceted crystals at nano-scale. (**E**–**H**) The cross-section morphology by TEM imaging showing the double-layer ultra-thin structure of nanocoating on the nichrome alloy surface: (**E**) overall TEM BF images, (**F** and **G**) zoom-in TEM BF images showing the detailed structure of inner amorphous Si–Cr–O layer and outer Cr_2_O_3_ layer, (**H**) corresponding SAED patterns of polycrystalline Cr_2_O_3_ layer and amorphous Si–Cr–O layer. (**I**) TEM-EDS mapping showing the element distribution on the cross-section of this surface nanocoating, in which the white dash line indicates the position of oxidation coating.

**Figure 9:**
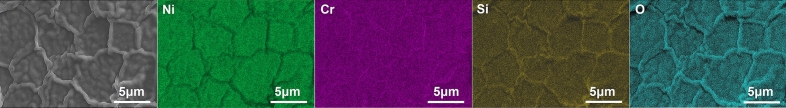
EDS mapping showing the non-uniform element distribution on surface triple hierarchical nanocoating of nichrome alloy after ENP processing. Cr exhibits an almost indiscriminate distribution. The raised portion of folds, enriched in Si and O but lacking Ni, are possibly the GBs with a greater degree of oxidation. This hierarchical oxidation coating should be Ni-depleted, and those well-faceted nanocrystals should be almost chromium oxide (Cr_2_O_3_).

**Figure 10:**
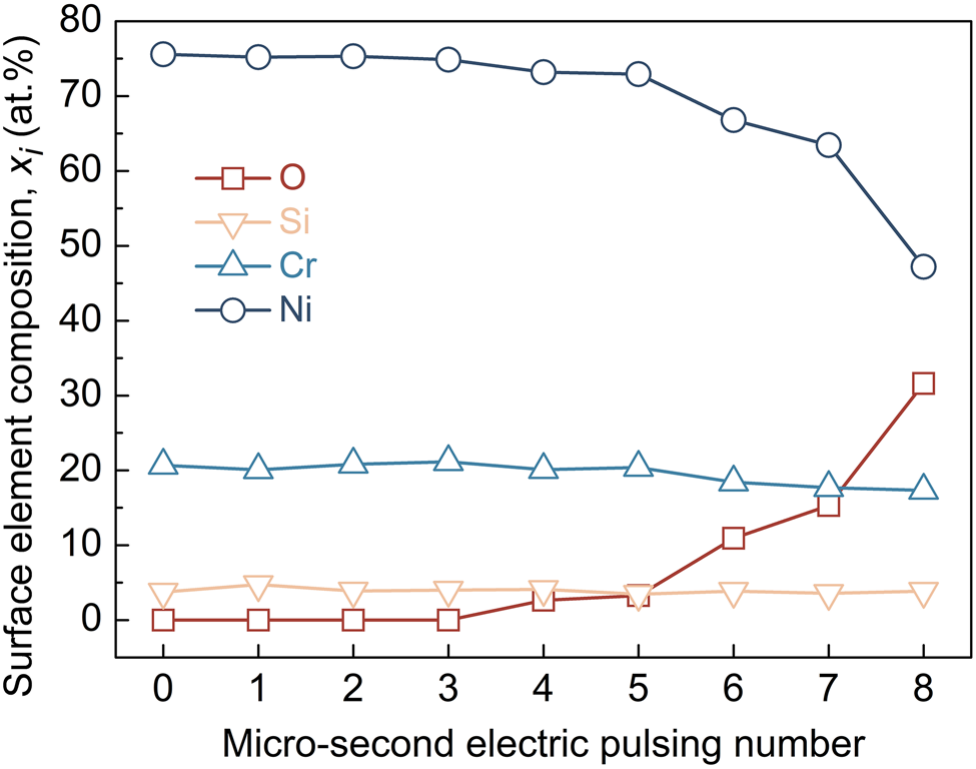
EDS measurements showing the evolution of element concentration on the surface triple hierarchical nanocoating of nichrome alloy as a function of pulsing number. The ENP processing has the current density of 3.15 × 10^10^ A/m^2^, the pulsing duration of 1 μs and the pulsing frequency of 100 kHz. Here pulsing number is corresponding to pulsing cycle, which has 10 μs processing time with 1 μs pulsing time in it. With the ENP processing ongoing, oxygen content gradually increases whilst nickel becomes depleted. Silicon and chromium stay almost constant in this case, indicating that it is mainly the chromium oxide and silicon oxide formation on nichrome alloy surface.

**Figure 11:**
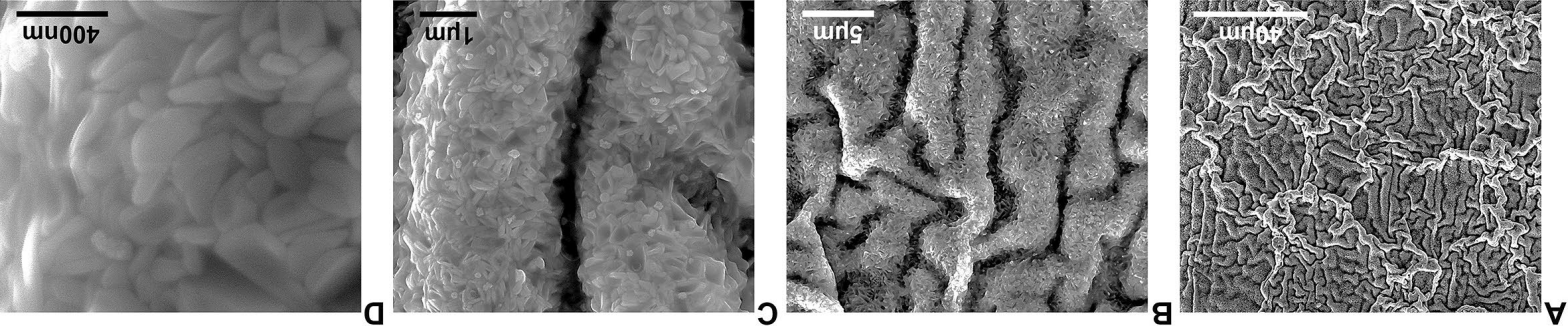
The exterior surface morphology by SEM imaging showing the coarse surface structure on nichrome alloy under conventional furnace at 1400 °C (melting point) for ten seconds. With the magnification increasing from figure (**A**) to figure (**D**), the spheroidization and coarsening of surface layer is clearly observed compared with ENP treated nichrome alloy, which can lead to the property degradation.

The rapid heating speed by this high-intensity ENP technology in air atmosphere can activate the altered oxidation behavior of the nichrome alloy, forming a unique oxidation coating on surface (Fig. 7). Generally, in the oxidation of nichrome alloys, loose NiO and Cr_2_O_3_ are preferentially formed and finally transformed into NiCr_2_O_4_ spinel in the case of prolonged oxidation^63^. It is interesting that, with the ENP processing, a dense chromium oxide nanocoating with triple hierarchical structure on alloy exterior surface can be obtained after eight consecutive pulses are released. This rough coating surface displays a fold-like morphology at micro-scale (Fig. 8A,B) and the zoom-in image shows some sub-nanoscale ravines inside (Fig. 8C). The inspection at higher resolution further shows that this surface coating is composed of the stacking of massive well-faceted nanocrystals (< 200 nm) (Fig. 8D). As an outcome of instantaneous surface localized modification, this dense hierarchical micro-nano structure on exterior surface conceivably possesses high hardness and specific surface area, which may find many applications in wear-resistant and gas sensors. It should be noted that the proposed ENP coating technology can be conducted in various gas environments with different conductive alloys as substrate, thereby fabricating surface ceramic layers or even thin films flexibly. The composition by EDS measurement (Figs. 9, 10) further clarifies that this surface oxidation coating mainly contains the chromium oxide, while almost no formation of nickel-containing oxides is involved during ENP processing. In fact, this non-equilibrium structure stimulated by the ENP processing is completely unobtainable under conventional thermal method at 1400 °C (melting point) for ten seconds (Fig. 11), which only leads to roughened and loose surface layer with the hardness decreasing and the tribological properties degradation^64^. The spheroidization and coarsening of crystals observed will also damage their electrical and gas-sensitive properties^65^.

**Figure 12:**
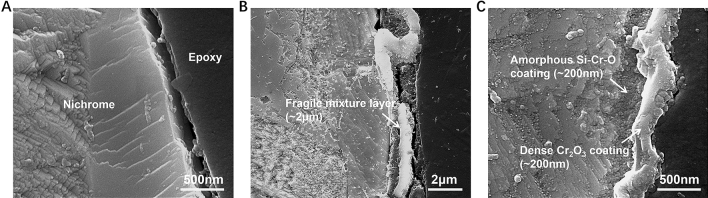
SEM images showing the cross-section view of surface oxidation coating on nichrome alloy wires etched by HCl/CuSO_4_ ethanol solution. (**A**) Raw sample. (**B**) After conventional heating. (**C**) After ENP processing. The ENP processing has the current density of 3.15 × 10^10^ A/m^2^, the pulsing duration of 1 μs and the pulsing frequency of 100 kHz. Nichrome alloy can hardly form any oxidation layer on its surface under natural conditions at room temperature. Conventional heating at 1400 °C leads to the appearance of fragile layer as expected. Excessive high temperature oxidation is generally not welcomed in metallic materials due to material loss and property damage. Indeed, the thermal oxidation of nichrome alloy by prolonged conventional heating brings unwanted Ni/Cr loss and a thick (~ 2 μm) irregular layer that significantly spalled from surface. In contrast, a dense double-layer coating with a thickness of nanometer scale is achieved by this ENP processing.

**Figure 13:**
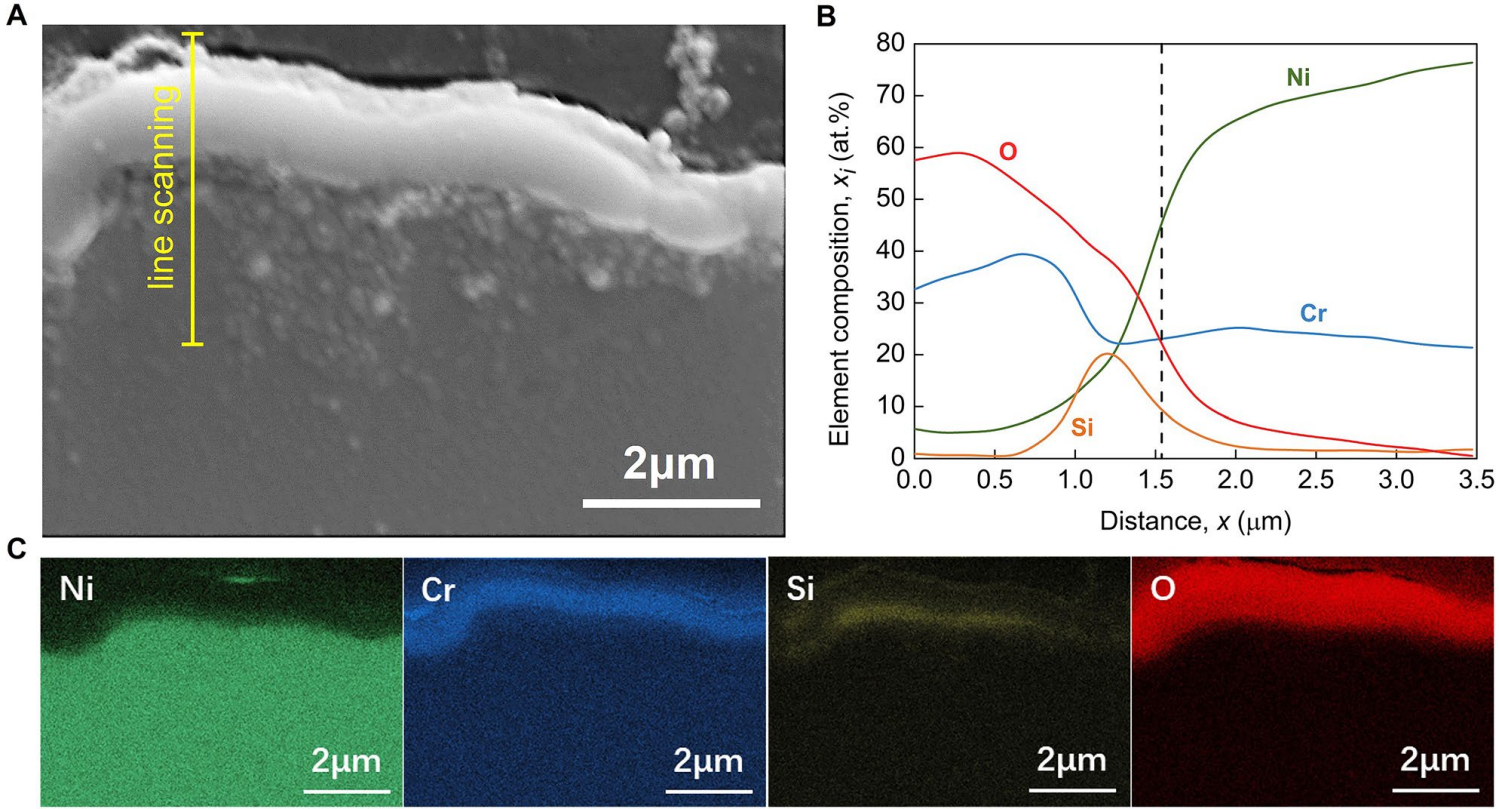
Cross-section EDS measurements on the surface of nichrome alloy under conventional furnace at 1400 °C (melting point) for ten seconds. (**A**) SEM image showing the location for EDS measurements. (**B**) EDS line scanning showing the various elements presented inside the surface oxidation layer. (**C**) EDS element mapping showing the formation of single-layered Cr_2_O_3_/NiO/SiO_2_ mixture on surface.

The cross-section morphology of this nanocoating by quasi-instantaneous ENP processing is given in Fig. 12, with a comparison to that by conventional heating. The mounted nichrome alloys are etched by 10%HCl/3%CuSO_4_ ethanol solution to enhance the visualization of nanocoating. It should be Cr_2_O_3_/NiO/SiO_2_ mixture formed in alloy by conventional furnace (Fig. 13). Studies find that long-time thermal heating, even at lower temperatures, can generate the surface mixed oxides and the matrix intergranular oxides^63,66^. However, as shown in Fig. 12C, a dense double-layer composite coating with a thickness of about half a micrometer on nichrome alloy surface can be achieved by the ENP processing. Its transient nature can be one explanation for the formation of ultra-thin Cr_2_O_3_ coatings (~ 200 nm) because of kinetically insufficient time for crystal growth. Yet, ENP processing surprisingly produces not only a denser Cr_2_O_3_ coating but also one extra ultra-thin amorphous Si–Cr–O nanocoating beneath it (Fig. 8H). This double-layer ultra-thin coating covers the nichrome alloy well, without excessive detrimental oxygen intergranular diffusion into alloy matrix (Fig. 8E,I). High-intensity ENP processing not only promotes the formation of Si/Cr-contained oxidation coating by enhancing diffusion, but also enlarges the diffusivities of Cr and Si in varying degrees^67^. Therefore, this separated double-layered nanocoating can be obtained on localized alloy surface in microseconds by ENP processing, which is required a long critical time by conventional thermal methods^68,69^. Our experiments demonstrate the potential of ENP processing for a rapid development of protective coatings. Some studies find the preferred formation of unwanted NiO oxide on Ni–Cr based superalloy surface after short-term heat exposure^68,70^, which does not have necessary protective function^71^. In our case, electropulsing produces thin protective Cr_2_O_3_ layer strongly adhered to the alloy matrix. The folded structure of the coating layer indicates that despite high temperature reached during ENP processing, grain boundary diffusion still has main contribution into the mass transport^72^. All these outcomes are undoubtedly from the enhanced field effects produced by this unique ENP processing. Such adherent ultra-thin double-layered coating with well-bonded and defect-free interface with alloy matrix (Fig. 8F,G) should have good anti-corrosion protection ability^71^ and bring this nichrome alloy with better room temperature gas sensitivity and mechanical properties^73,74^.

## Discussion

By electric nano pulsing (ENP) technology, the massive electric energy released into polycrystalline materials within the micro-nano seconds enables the extremely rapid Joule heating effects and the ultra-strong electric field effects, bringing the intense non-equilibrium microstructure evolutions at nanometer spatial scale and nanosecond temporal scale, which cannot be achieved through conventional thermal treatment. Potential applications of this high-intensity ENP technology can be excavated in the related industrial fields, towards an upgraded high-rate manufacturing in micro-nano seconds’ level. ENP not only opens a new technical avenue for the quasi-instantaneous materials processing by electrically assisted treatment, but also contributes to the refinement of field-assisted materials processing via exploring the application or mechanism of relevant field effects.

The feature of instantaneous treatment can promote the potential of the ENP technology for versatile and efficient microstructure processing of alloys, because the samples can be easily attached to the fixed electrodes to achieve the continuous ENP processing. The highly compatible electrodes in the developed ENP device can be designed as flexible ones to adapt to the instantaneous processing of complex-shaped parts. They can even be designed to load multiple samples at once for the high-throughput ENP processing in multi-tasking scenarios. In addition, ENP should be a scientific tool to explore the intrinsic mechanism of field effects on its interaction with microstructure in materials. It allows a finely controllable electropulsing profile such that it can be applied for the instantaneous stimulation of desired field effects in a tunable manner. The exclusive study of field effects requires to de-couple them from the outcomes by Joule heating. Just by releasing few single electric nano pulses, its high intensity nature ensures the significant non-thermal effects whilst its short duration nature ensures the reduced influences by thermal effects. Like other electropulsing technologies, ENP can be used for nanopowders synthesis by electric explosion or for the capacitive discharge welding by joint connection with high efficiency and short processing time. Due to its ultra-fast and heavily non-equilibrium nature, ENP can also be used for improving the strength-plasticity synergy in engineering materials by promoting grain refinement and GBs reconstruction. ENP can be extended to process a wide range of non-conductive materials by inserting conductive parts, like graphite felt, to obtain their instantaneous thermal treatment. By this, ENP can even be coupled with novel sintering method to develop ENP-based flash sintering or ENP-based ultra-fast high-temperature sintering that achieves instantaneous consolidation of powders within micro-nano seconds.

## Conclusion

For the first time, extraordinary electropulsing technology of electric nano pulsing has been introduced to realize the quasi-instantaneous processing on the localized microstructures in materials, which achieves ultra-high voltage (up to 1000 V), ultra-high current density (up to 10^11^–10^12^ A/m^2^), ultra-short pulse duration (less than 1 μs) and ultra-high pulsing frequency (up to 100 kHz) simultaneously. The massive electric energy released into polycrystalline materials within the micro-nano seconds enables the extremely rapid Joule heating effects and the ultra-strong electric field effects, bringing the intense non-equilibrium microstructure evolutions at nanometer spatial scale and nanosecond temporal scale, including surface nanocoating and defect reconfiguration. They absolutely cannot be achieved through conventional thermal treatment. Potential applications of this high-intensity ENP technology can be excavated in the related industrial fields, towards an upgraded high-rate manufacturing in micro-nano seconds’ level. ENP not only opens a new technical avenue for the quasi-instantaneous materials processing by electrically assisted treatment, but also contributes to the refinement of electropulsing treatment for exploring the application or mechanism of relevant field effects (Supplementary Movies S1, S2).

### Supplementary Information

Supplementary Legends.

Supplementary Video 1.

Supplementary Video 2.

## Data Availability

All data generated or analyzed during this study are included in this published article (and its Supplementary Information files).
