# Supplementary material for: Quasi-instantaneous materials processing technology via high-intensity electrical nano pulsing
Source: Sci Rep. 2024 Jan 3;14:434. doi: 10.1038/s41598-023-50698-w (PMC10764874; doi:10.1038/s41598-023-50698-w)
Supplement: Supplementary file 1 — Supplementary Legends. [file 41598_2023_50698_MOESM1_ESM.docx]

Supplementary Materials for

**Quasi-instantaneous Materials Processing Technology via High-intensity Electrical Nano Pulsing**

Eugene A. Olevsky, Runjian Jiang, Wenwu Xu, Andrii Maximenko, Thomas Grippi, Elisa Torresani

**This PDF file includes:**

Movies S1 to S2

**Movie S1.** Nichrome alloy wire captured by infrared camera under the ENP processing with eight consecutive pulses at the current density of 3.15×10^10^A/m^2^, the pulsing duration of 1μs and the pulsing frequency of 100KHz. The temperature raise is obvious and is estimated to reach the melting point by simulation.^1^

**Movie S2.** Nichrome alloy wire captured by infrared camera under the ENP processing with two consecutive pulses at the current density of 6.98×10^10^A/m^2^, the pulsing duration of 1μs and the pulsing frequency of 100KHz. The temperature raise is obvious and is estimated to reach the melting point by simulation.^1^

^1^The infrared camera (Teledyne FLIR A700) was used in the temperature measurement during ENP processing. It is noted that this infrared camera has a time frame of 30Hz (~33ms per sampling) and temperature interval of -120^o^C to +2000^o^C, which is applicable for the observation of temperature raise but not sufficiently advanced for the detection of temperature change at nano-second scale. It seems that no commercial infrared camera with time frame higher than 1MHz can be found in the market, therefore the temperature evolution during ENP processing should be predicted by combining simulation and experimental observation.
